# Metabolic profiles characterizing different phenotypes of polycystic ovary syndrome: plasma metabolomics analysis

**DOI:** 10.1186/1741-7015-10-153

**Published:** 2012-11-30

**Authors:** Yue Zhao, Li Fu, Rong Li, Li-Na Wang, Yan Yang, Na-Na Liu, Chun-Mei Zhang, Ying Wang, Ping Liu, Bin-Bin Tu, Xue Zhang, Jie Qiao

**Affiliations:** 1 Reproductive Medical Center, Department of Obstetrics and Gynecology, Peking University Third Hospital, Beijing, China; 2 Key Laboratory of Assisted Reproduction, Ministry of Education, Beijing, China; 3 Beijing Key Laboratory of Reproductive Endocrinology and Assisted Reproductive Technology, Beijing, China

**Keywords:** polycystic ovary syndrome, amino acid metabolism, carbohydrate and lipid metabolism, insulin resistance, inflammation

## Abstract

**Background:**

Polycystic ovary syndrome (PCOS) is a heterogeneous endocrine disorder accompanied with an increased risk of developing type 2 diabetes mellitus and cardiovascular disease; despite being a common condition, the pathogenesis of PCOS remains unclear. Our aim was to investigate the potential metabolic profiles for different phenotypes of PCOS, as well as for the early prognosis of complications.

**Methods:**

A total of 217 women with PCOS and 48 healthy women as normal controls were studied. Plasma samples of subjects were tested using two different analytical platforms of metabolomics: ^1^H nuclear magnetic resonance (NMR) and gas chromatography/time-of-flight mass spectrometry (GC/TOF-MS).

**Results:**

Our results showed that carbohydrate, lipid and amino acid metabolisms were influenced in PCOS. The levels of lactate, long-chain fatty acids, triglyceride and very low-density lipoprotein were elevated, while glucose, phosphatidylcholine and high-density lipoprotein (HDL) concentrations were reduced in PCOS patients as compared with controls. Additionally, the levels of alanine, valine, serine, threonine, ornithine, phenylalanine, tyrosine and tryptophan were generally increased, whereas the levels of glycine and proline were significantly reduced in PCOS samples compared to controls. Furthermore, the ratio of branched-chain amino acid to aromatic amino acid concentrations (BCAA/AAA) in PCOS plasma was significantly reduced in PCOS patients and was insusceptible to obesity and insulin sensitivity.

**Conclusions:**

Our results suggested that the enhanced glycolysis and inhibited tricarboxylic acid cycle (TAC) in women with PCOS. Decrease of BCAA/AAA ratio was directly correlated with the development of PCOS. Ovulatory dysfunction of PCOS patients was associated with raised production of serine, threonine, phenylalanine, tyrosine and ornithine. Elevated levels of valine and leucine, and decreased concentrations of glycine in PCOS plasma could contribute to insulin sensitivity and could be considered as the potential biomarkers for long-term risk assessment of diabetes mellitus.

## Background

Polycystic ovary syndrome (PCOS) is a complex and heterogeneous endocrine disorder with well established metabolic abnormalities. The prevalence of PCOS is approximately 5% to 10% in reproductive-age women [1]. Hyperandrogenism, oligomenorrhea, chronic anovulation, and hyperinsulinemia are common clinical manifestations of PCOS [2]. Women with this syndrome have an increased risk of developing type 2 diabetes mellitus (DM2) and cardiovascular disease (CVD) [1,3,4]. The pathogenesis of PCOS remains a mystery, although it is considered as a polygenic trait that might result from the interaction of susceptible genomic variants and environmental factors [5,6].

At present, alterations in several metabolic pathways have been implicated in the pathophysiology of PCOS, including abnormalities in steroid hormone regulation and insulin signaling pathway [7-9]. Moreover, there is increasing focus on the complications associated with metabolic disturbances among women with PCOS. Obesity, insulin resistance, dyslipidemia and inflammation have been recognized as risk factors for developing DM2 and CVD in PCOS [10,11]. This highlights the need to understand the metabolic dysfunction in PCOS for prevention of long-term complications through appropriate screening, diagnosis and intervention.

In addition, the diagnosis of PCOS has also been a controversial issue [12]. Following the 2003 Rotterdam criteria [13], PCOS can be divided into four different phenotypes: (A) hyperandrogenism, chronic anovulation and polycystic ovaries (HA+AO+PCO); (B) chronic anovulation and polycystic ovaries but no clinical or biochemical hyperandrogenism (AO+PCO); (C) hyperandrogenism and chronic anovulation but normal ovaries (HA+AO); (D) hyperandrogenism and polycystic ovaries but ovulatory cycles (HA+PCO). However, it is not clear whether routine characteristics of metabolic abnormality markers could be found in all these phenotypes.

The systemic changes that occur in PCOS reflect not only changes in ovarian function, but also changes in whole-body metabolism. Metabolomics enables the characterization of endogenous small molecules that serve as direct profiles of biochemical activity and correlate with the phenotype [14]. As compared with genomics, transcriptomics and proteomics, metabolomics provides the most predictive biomarkers of this disease. In order to investigate potential metabolic profiles for different phenotypes of PCOS, as well as for the early prognosis of complications, we conducted a plasma metabolomic analysis to investigate the metabolic changes underlying different phenotypes of PCOS using both ^1^H nuclear magnetic resonance (NMR) and gas chromatography/time-of-flight mass spectrometry (GC/TOF-MS); these demonstrated to be complementary analytical technologies [15].

## Methods

### Study populations

This randomized study population consisted of 217 PCOS patients and 48 women of similar age as controls, who visited the Division of Reproductive Center, Peking University Third Hospital, from March 2010 to March 2011. According to 2003 Rotterdam criteria, the diagnostic traits of PCOS are the presence of two or more of: oligo-ovulation and/or anovulation, clinical and/or biochemical signs of hyperandrogenism, and polycystic ovaries after exclusion of other etiologies (congenital adrenal hyperplasia, androgen-secreting tumors, Cushing's syndrome, 21-hydroxylase-deficient non-classic adrenal hyperplasia, androgenic/anabolic drug use or abuse, thyroid dysfunction, hyperprolactinemia, type 2 diabetes mellitus and cardiovascular disease). Women who had received any hormonal treatment or insulin-lowering agent during the last 3 months were excluded from the study. The control subjects were selected from women attending the clinic on account of male azoospermia. All controls had regular menstrual cycles and normal androgen levels. This study was approved by the Ethics Committee of Peking University Third Hospital. Informed consent was obtained from all participants prior to inclusion in this study.

### Sample preparation and metabolomic assays

Venous blood (3 ml) was collected into a heparin sodium tube and the plasma was collected by centrifugation. Each plasma sample was split into two aliquots and run in parallel using two different analytical platforms, as described previously [16,17].

For the ^1^H NMR measurement, an aliquot of 300 μl of plasma was mixed with 250 μl D_2_O and 50 μl 3-trimethylsilyl-^2^H_4_-propionic acid sodium salt (TSP) in D_2_O (1 mg/ml) in a 5 mm NMR tube. The D_2_O provided a field-frequency lock solvent for the NMR spectrometer and the TSP served as an internal reference of chemical shift. ^1^H NMR spectra of the plasma samples were acquired on a Varian INOVA 600 MHz NMR spectrometer at 27 °C by using Carr-Purcell-Meiboom-Gill (CPMG) spin-echo pulse sequence with a total spin-spin relaxation delay (2nτ) of 320 ms. The free induction decays (FIDs) were collected into 32K data points with a spectral width of 8,000 Hz and 64 scans. The FIDs were zero-filled to double size and multiplied by an exponential line-broadening factor of 0.5 Hz prior to Fourier transformation (FT). In addition, diffusion-edited experiments were also carried out with bipolar pulse pair-longitudinal eddy current delay (BPP-LED) pulse sequence. The gradient amplitude was set at 35.0 G/cm, with a diffusion delay of 100 ms. A total of 128 transients and 16K data points were collected with a spectral width of 8,000 Hz. A line-broadening factor of 1 Hz was applied to FIDs before Fourier transformation. All plasma ^1^H NMR spectra were manually phased and baseline-corrected using VNMR 6.1C software (Varian, Inc.). For CPMG spectra, each spectrum over the range of δ 0.4 to 4.4 was data reduced into integrated regions of equal width (0.01 ppm). For BPP-LED data, each spectrum over the range of δ 0.1 to 6.0 was segmented into regions of equal width (0.01 ppm). The regions containing the resonance from residual water (δ 4.6 to 5.1) were excluded. The integral values of each spectrum were normalized to a constant sum of all integrals in a spectrum in order to reduce any significant concentration differences between samples. Identification of metabolites in spectra was accomplished based on information in the literature and the Chenomx NMR Suite 5.0 (Chenomx, Calgary, Canada).

For the GC/TOF-MS measurement, plasma samples (100 μl) were thawed before the immediate addition of 500 μl of methanol (100%) to stop enzymatic activity. The samples were vortexed thoroughly, and 20 μl of a ribitol stock solution (0.2 mg/ml) was added as an internal reference. The mixture was placed on a shaker at 70°C for 15 minutes and centrifuged at 10,000 *g *for 10 minutes. The supernatant was mixed with 500 μl of pure water and 250 μl of chloroform, and was centrifuged at 4,000 rpm for 15 minutes. The upper (polar) phase was separated and then evaporated to dryness under a stream of N_2 _gas in a thermostatically controlled water bath (60°C). Methoxyamine hydrochloride (20 μl, 20 mg/ml pyridine) was then added to the dried fraction of the polar phase. Following continuous shaking at 30°C for 90 minutes, 40 μl of *N*-methyl-*N*-trimethylsilyltrifluoroacetamide (MSTFA) was added, and the tube was incubated at 37°C for 30 minutes, then kept at room temperature for 120 minutes. Solutions (0.3 μl) were injected at a split ratio of 25:1 into a GC/TOF-MS system consisting of an HP 6890 gas chromatograph and a time-of-flight mass spectrometer (Waters Co., Milford, MA, USA) on a 30 m DB-5 column (250 μm inner diameter., 0.25 μm film; Agilent Technologies, Palo Alto, CA, USA). The injection temperature was 230°C, the interface temperature was set to 290°C, and the ion source temperature was adjusted to 220°C, with electron energy of 70 eV. Helium was set to a column flow rate of 1 ml/min. After a 5-minute solvent delay time at 70°C, the oven temperature was increased to 310°C in increments of 5°C/min, followed by a 1-minute isocratic cool down to 70°C and an additional 5-minute delay. MassLynx software (Waters Co.) was used to acquire the chromatographs. NIST02 libraries with electron impact (EI) spectra were searched rigorously for all the peaks detected with the total ion current (TIC), to identify the metabolites. Compounds were also identified by comparison of their mass spectra and retention times with those of commercially available reference compounds. The integral values of each spectrum were normalized to the integral of ribitol stock in order to reduce any significant concentration differences between samples.

### Data analysis

Multivariate pattern recognition analysis was carried out by using SIMCA-P plus software (V. 10.0). Principal component analysis (PCA) was performed for data from different groups to detect the distributions and separations among those groups. Prior to PCA, all data variables were mean centered and preprocessed using orthogonal signal correction (OSC) to remove variations from non-correlated factors such as the instability of the spectrometer, inconstancy in sample preparation, and variability of some metabolites depending on the subject.

Multiple comparisons of the measured metabolite intensities of PCOS subgroups and the control group were made by one-way analysis of variance (ANOVA) analysis using SPSS V. 19 (SPSS, Chicago, IL, USA), and the adjustment methods was the Bonferroni correction. Values were presented as mean ± SD, and an adjusted *P *value <0.05 was considered statistically significant. Associations between the specific metabolites and PCOS controlling for age, body mass index (BMI) and insulin resistance (IR) were tested by linear regression analysis (SPSS V. 19).

## Results

### Baseline characteristics

Of 217 PCOS patients according to the Rotterdam guidelines, they were 72 patients (33.2%) presented with the classic PCOS phenotype (HA+AO+PCO); 74 patients (34.1%) with chronic anovulation and polycystic ovaries but no clinical or biochemical hyperandrogenism (AO+PCO); 33 patients (15.2%) with hyperandrogenism and chronic anovulation but normal ovaries (HA+AO); 38 patients (17.5%) with hyperandrogenism and polycystic ovaries but ovulatory cycles (HA+PCO). Baseline characteristics for patients with different phenotypes of PCOS and controls were described in Table 1. A broad spectrum of metabolic and biochemical changes in PCOS subgroups compared with the control group were indicated, including the obviously enhanced triglyceride and low-density lipoprotein (LDL) level and reduced HDL level, a marked augment in serum concentrations of androgens such as androstenedione, increased ratio of luteinizing hormone (LH) to follicle-stimulating hormone (FSH). Overall, these changes in PCOS patients were clinically associated with the phenotypes and complemented the metabolomic analysis.

**Table 1:** Baseline characteristics of four polycystic ovary syndrome (PCOS) phenotypes and control subjects

Characteristic	PCOS				Controls
		
	A (HA+AO+PCO)		C (HA+AO)	D (HA+PCO)	
Number	72 (33.2%)	74 (34.1%)	33 (15.2%)	38 (17.5%)	48
Age, years	28.00 ± 0.59	28.74 ± 0.45	28.03 ± 0.65	28.79 ± 0.53	29.78 ± 0.56
BMI, kg/m^2^	24.29 ± 0.46**	24.57 ± 0.54**	25.37 ± 0.81**	24.21 ± 0.67**	21.63 ± 0.36
LH, mIU/ml	11.58 ± 0.77**	7.57 ± 1.04**	6.47 ± 0.95**	7.10 ± 1.28**	3.95 ± 0.26
LH/FSH	1.74 ± 0.10**	1.18 ± 0.17**	1.09 ± 0.12**	1.10 ± 0.14**	0.54 ± 0.03
T, nmol/l	2.06 ± 0.09**	1.26 ± 0.06*	1.73 ± 0.13**	1.95 ± 0.16**	1.31 ± 0.10
A, nmol/l	16.07 ± 0.50**	7.98 ± 0.25*	14.32 ± 0.78**	17.18 ± 1.05**	6.55 ± 0.27
CHO, mmol/l	4.84 ± 0.09*	4.66 ± 0.10	4.85 ± 0.18	5.23 ± 0.18**	4.42 ± 1.32
TG, mmol/l	1.47 ± 0.09**	1.44 ± 0.10*	1.50 ± 0.17*	1.56 ± 0.12*	1.04 ± 0.13
HDL, mmol/l	1.23 ± 0.03**	1.17 ± 0.03**	1.20 ± 0.05*	1.29 ± 0.05	1.38 ± 0.04
LDL, mmol/l	2.93 ± 0.08**	2.88 ± 0.10*	3.03 ± 0.17*	3.13 ± 0.16**	2.47 ± 0.13

Data are presented as mean ± SD. *P <0.01; **P <0.001. A = androstenedione; AO = anovulation; BMI = body mass index; CHO = cholesterol; FSH = follicle-stimulating hormone; HA = hyperandrogenism; HDL = high-density lipoprotein; LDL = low-density lipoprotein; LH = luteinizing hormone; PCO = polycystic ovaries; TG = triglyceride.

### Aberrance of carbohydrate and lipid metabolism in PCOS

**Figure 1:**
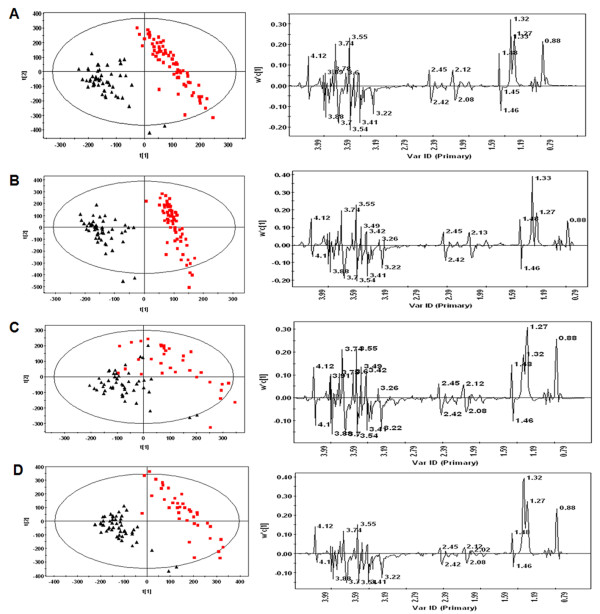
**^1^H nuclear magnetic resonance (NMR) analysis**. Orthogonal signal correction/principal component analysis (OSC-PCA) scores plot (left column) and loadings plots (right column) for Carr-Purcell-Meiboom-Gill (CPMG) ^1^H-NMR spectra of plasma from the four phenotypes of polycystic ovary syndrome (PCOS) patients (red squares) and controls (black triangles). **(A) **PCOS patients with hyperandrogenism (HA), anovulation (AO) and polycystic ovaries (PCO) (HA+AO+PCO) vs controls; **(B) **PCOS patients with anovulation and polycystic ovaries (AO+PCO) vs controls; **(C) **PCOS patients with hyperandrogenism and anovulation (HA+AO) vs controls; **(D) **PCOS patients with hyperandrogenism and polycystic ovaries (HA+PCO) vs controls.

The plasma samples from the PCOS and control groups were analyzed using ^1^H NMR spectroscopy. OSC-PCA was performed to evaluate the population structure of each PCOS subgroup comparing to the control group (Figure 1). The metabolites responsible for the differences among the various groups were identified and summarized in Table 2. The results showed that all of the four PCOS phenotypes had higher levels of very low-density lipoprotein (VLDL), LDL, fatty acids, unsaturated fatty acids and an unidentified sugar (δ 3.74 ppm), but had lower levels of phosphatidylcholine and lysyl-albumin as compared with the control group, respectively, which indicated the aberrance of lipid metabolism in PCOS. Specially, elevated level of lactate and reduced level of glucose in PCOS phenotypes except the feature (HA+AO) showed the carbohydrate metabolic disorder in the patients with polycystic ovaries.

**Table 2:** Changes in the relative levels of metabolites in polycystic ovary syndrome (PCOS) phenotypes compared with the control group based on the Carr-Purcell-Meiboom-Gill (CPMG) and bipolar pulse pair-longitudinal eddy current delay (BPP-LED) ^1^H nuclear magnetic resonance (NMR) spectra

Chemical shift, ppm	Identified metabolites	Control group	A group (HA+AO+PCO)		B group (AO+PCO)		C group (AO+HA)		D group (HA+PCO)	
			
			Mean ± SD	*P *adjusted	Mean ± SD	*P *adjusted	Mean ± SD	*P *adjusted	Mean ± SD	*P *adjusted
CPMG										
0.84 to 0.90, 1.26 to 1.30	Lipoprotein	0.09 ± 0.003	0.12 ± 0.005	<0.001 U	0.10 ± 0.005	0.044 U	0.12 ± 0.005	0.005 U	0.13 ± 0.005	<0.001 U
1.31 to 1.34, 4.09 to 4.14	Lactate	0.13 ± 0.004	0.15 ± 0.003	0.006 U	0.15 ± 0.004	0.006 U	0.14 ± 0.005	1.000 -	0.16 ± 0.005	0.001 U
1.46 to 1.48	Alanine	0.018 ± 0.004	0.019 ± 0.003	1.000 -	0.018 ± 0.004	1.000 -	0.019 ± 0.004	1.000 -	0.019 ± 0.004	1.000 -
3.40 to 4.00	Glucose	0.44 ± 0.05	0.40 ± 0.05	0.001 D	0.41 ± 0.05	0.013 D	0.42 ± 0.06	1.000 -	0.39 ± 0.06	0.001 D
3.74	U	0.009 ± 0.002	0.013 ± 0.002	<0.001 U	0.013 ± 0.002	<0.001 U	0.012 ± 0.003	<0.001 U	0.013 ± 0.002	<0.001 U
BPP-LED										
0.9,0.86, 1.26,1.3,1.34	VLDL/LDL	0.25 ± 0.04	0.28 ± 0.05	0.009 U	0.27 ± 0.05	0.045 U	0.27 ± 0.06	0.032 U	0.29 ± 0.05	0.008 U
1.22	HDL	0.051 ± 0.005	0.048 ± 0.006	0.025 D	0.050 ± 0.007	1.000 -	0.050 ± 0.006	1.000 -	0.047 ± 0.007	0.047 D
1.58	Lipid- CH2CH2CO	0.012 ± 0.003	0.014 ± 0.003	0.018 U	0.013 ± 0.003	0.453 -	0.014 ± 0.005	0.276 -	0.014 ± 0.004	0.005 U
2.06	NAc	0.018 ± 0.001	0.018 ± 0.001	1.000 -	0.017 ± 0.002	0.105 -	0.017 ± 0.001	0.012 D	0.017 ± 0.001	0.001 D
2.22	FA	0.59 ± 0.96	0.72 ± 0.17	<0.001 U	0.73 ± 0.19	<0.001 U	0.80 ± 0.22	<0.001 U	0.82 ± 0.18	<0.001 U
2.02, 2.74, 5.18 to 5.3	UFA	0.071 ± 0.005	0.078 ± 0.007	<0.001 U	0.077 ± 0.008	<0.001 U	0.076 ± 0.009	0.049 U	0.077 ± 0.007	0.001 U
2.94 to 2.98	Lysyl-albumin	0.014 ± 0.002	0.011 ± 0.002	<0.001 D	0.012 ± 0.002	<0.001 D	0.012 ± 0.003	0.024 D	0.011 ± 0.002	<0.001 D
3.22, 3.26	PtdCho	0.027 ± 0.005	0.023 ± 0.005	<0.001 D	0.023 ± 0.005	<0.001 D	0.022 ± 0.005	0.001 D	0.022 ± 0.005	<0.001 D

The CPMG pulse sequence was used to emphasize the small metabolites in the plasma, while the BPP-LED pulse sequence presented only broad peaks from the macromolecules in samples. Data are presented as mean ± SD, and U (up) and D (down) next to numbers indicates the significant elevated or reduced concentration of metabolites (*P *<0.05), respectively. AO = anovulation; FA = fatty acid; HA = hyperandrogenism; HDL = high-density lipid; LDL = low-density lipid; NAc = *N*-acetyl groups of glycoproteins; PCO = polycystic ovaries; PtdCho = phosphatidylcholine; U = unidentified sugar; UFA = unsaturated fatty acid; VLDL = very low-density lipid.

**Figure 2:**
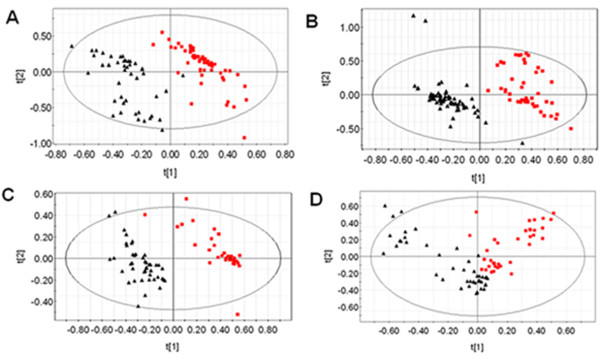
**Gas chromatography/time-of-flight mass spectrometry (GC/TOF-MS) analysis**. Orthogonal signal correction/partial least squares (OSC-PLS) scores plot of GC/TOF-MS data of plasma from the four phenotypes of polycystic ovary syndrome (PCOS) patients (red squares) and the controls (black triangles). **(A) **PCOS patients with hyperandrogenism (HA), anovulation (AO) and polycystic ovaries (PCO) (HA+AO+PCO) vs controls; **(B) **PCOS patients (AO+PCO) vs controls; **(C) **PCOS patients (HA+AO) vs controls; **(D) **PCOS patients (HA+PCO) vs controls.

However, a total of 45 plasma metabolites were identified as endogenous metabolites by GC/TOF-MS analysis, including amino acids, fatty acids, sugars, and organic acids. Multivariate statistical analysis was performed, and the OSC-partial least squares (PLS) scores plot illustrated that each PCOS phenotype exhibited obvious differences with the control group (Figure 2). Moreover, there were diverse significant differences were noted in the plasma levels of each PCOS phenotypes compared with the control group. The productions of lactate and long-chain fatty acids (for example, linoleic acid, palmic acid and stearic acid) were induced, whereas the production of glucose was inhibited in PCOS groups, which was consistent with ^1^H NMR data (Table 3).

**Table 3:** Changes in the relative levels of metabolites detected by gas chromatography/time-of-flight mass spectrometry (GC/TOF-MS) in the plasma of polycystic ovary syndrome (PCOS) and control subjects

Retention time, min	Identified metabolites	Control group	A group (HA+AO+PCO)		B group (AO+PCO)		C group (AO+HA)		D group (HA+PCO)	
			
			Mean ± SD	*P *adjusted	Mean ± SD	*P *adjusted	Mean ± SD	*P *adjusted	Mean ± SD	*P *adjusted
6.249	Lactate	2.17 ± 0.27	2.29 ± 0.46	0.039 U	2.5 ± 0.54	0.026 U	2.03 ± 0.61	0.079 -	2.82 ± 0.77	0.035 U
25.818	Glucose	4.96 ± 0.78	4.03 ± 0.32	0.019 D	4.02 ± 0.41	0.034 D	4.29 ± 0.62	0.054 -	4.27 ± 0.78	0.04 D
26.184	Galactose	1.94 ± 0.29	1.76 ± 0.13	0.582 -	1.69 ± 0.13	0.45 -	1.87 ± 0.43	1.000 -	1.87 ± 0.4	1.000 -
28.818	Palmic acid	0.72 ± 0.12	0.76 ± 0.12	0.876 -	0.91 ± 0.11	0.001 U	0.95 ± 0.15	<0.001 U	0.82 ± 0.13	0.014 U
29.751	Uric acid	0.27 ± 0.13	0.58 ± 0.17	<0.001 U	0.30 ± 0.21	1.000 -	0.16 ± 0.15	0.088 -	0.31 ± 0.18	1.000 -
31.785	Linoleic acid	0.05 ± 0.03	0.1 ± 0.09	0.003 U	0.36 ± 0.18	0.001 U	0.5 ± 0.21	<0.001 U	0.15 ± 0.16	0.019 U
32.418	Stearic acid	0.85 ± 0.14	0.91 ± 0.08	0.17 -	0.98 ± 0.08	0.023 U	0.96 ± 0.12	0.067 U	0.94 ± 0.10	0.01 U
45.636	Cholesterol	0.72 ± 0.35	0.69 ± 0.33	0.043 D	0.68 ± 0.26	0.038 D	0.80 ± 0.34	0.805 -	0.96 ± 0.73	0.342 -
7.149	Alanine	0.43 ± 0.14	0.56 ± 0.1	<0.001 U	0.63 ± 0.11	0.001 U	0.61 ± 0.6	<0.001 U	0.48 ± 0.17	0.813 -
9.866	Valine	0.46 ± 0.07	0.52 ± 0.1	<0.001 U	0.57 ± 0.07	0.001 U	0.53 ± 0.11	0.001 U	0.5 ± 0.08	0.042 U
11.3	Leucine	0.28 ± 0.09	0.28 ± 0.06	1.000 -	0.29 ± 0.09	1.000 -	0.32 ± 0.07	0.399 -	0.37 ± 0.11	0.002 U
11.85	Isoleucine	0.19 ± 0.08	0.18 ± 0.06	0.195 -	0.19 ± 0.05	1.000 -	0.18 ± 0.07	1.000 -	0.12 ± 0.05	0.001 D
11.933	Proline	0.59 ± 0.07	0.28 ± 0.08	<0.001 D	0.25 ± 0.08	0.001 D	0.26 ± 0.08	0.001 D	0.19 ± 0.07	0.001 D
12.15	Glycine	0.59 ± 0.09	0.49 ± 0.08	0.004 D	0.53 ± 0.08	0.026 D	0.50 ± 0.09	0.008 D	0.47 ± 0.11	0.001 D
13.6	Serine	0.29 ± 0.08	0.38 ± 0.07	<0.001 U	0.38 ± 0.07	<0.001 U	0.35 ± 0.09	0.012 U	0.24 ± 0.08	0.015 D
14.233	Threonine	0.35 ± 0.11	0.42 ± 0.08	<0.001 U	0.42 ± 0.08	0.001 U	0.39 ± 0.1	0.085 U	0.27 ± 0.09	0.006 D
16.317	Aspartate	0.13 ± 0.09	0.13 ± 0.09	1.000 -	0.2 ± 0.14	0.005 U	0.12 ± 0.06	1.000 -	0.08 ± 0.07	1.000 -
19.867	Phenylalanine	0.10 ± 0.05	0.24 ± 0.08	<0.001 U	0.21 ± 0.08	0.001 U	0.19 ± 0.08	0.001 U	0.11 ± 0.05	1.000 -
24.118	Ornithine	0.13 ± 0.08	0.25 ± 0.09	<0.001 U	0.23 ± 0.09	0.001 U	0.2 ± 0.1	0.002 U	0.12 ± 0.08	1.000 -
26.334	Lysine	0.28 ± 0.9	0.5 ± 0.18	<0.001 U	0.39 ± 0.11	0.126 -	0.34 ± 0.10	1.000 -	0.33 ± 0.26	1.000 -
26.634	Tyrosine	0.09 ± 0.04	0.24 ± 0.06	0.001 U	0.2 ± 0.07	0.001 U	0.17 ± 0.07	0.036 U	0.14 ± 0.07	1.000 -
31.7	Tryptophan	0.02 ± 0.01	0.17 ± 0.04	0.001 U	0.09 ± 0.06	0.003 U	0.05 ± 0.04	0.03 U	0.06 ± 0.05	0.002 U
	Endogenous AAs	3.8 ± 0.71	4.37 ± 0.62	0.001 U	4.32 ± 0.64	0.001 U	4.0 ± 0.73	0.294 -	3.38 ± 0.78	0.153 -
	Gluconeogenic AAs	3.4 ± 0.62	3.93 ± 0.52	0.003 U	4.14 ± 0.67	<0.001 U	3.6 ± 0.70	1.000 -	2.92 ± 0.65	0.002 D
	BCAA	0.87 ± 0.2	0.95 ± 0.22	1.000 -	1.03 ± 0.17	0.024 U	1.04 ± 0.20	0.017 U	0.98 ± 0.17	0.865 -
	AAA	0.21 ± 0.1	0.66 ± 0.17	0.001 U	0.49 ± 0.17	0.001 U	0.4 ± 0.16	0.019 U	0.31 ± 0.17	1.000 -
	BCAA/AAA	4.82 ± 1.8	1.51 ± 0.37	<0.001 D	2.35 ± 0.91	<0.001 D	3.09 ± 1.62	0.002 D	4.3 ± 2.50	0.298 -

Data are presented as mean ± SD, and U (up) and D (down) next to numbers indicates the significant elevated or reduced concentration of metabolites (*P *<0.05). Endogenous amino acids include all amino acids except for ornithine; gluconeogenic amino acids include alanine, valine, isoleucine, proline, glycine, serine, threonine, aspartate, phenylalanine, lysine and tyrosine; branched-chain amino acids (BCAA) include leucine, valine and isoleucine; aromatic amino acids (AAA) include phenylalanine, tyrosine and tryptophan. AO = anovulation; HA = hyperandrogenism; PCO = polycystic ovaries.

### Metabolic disturbance of amino acids in PCOS

Amino acids play important roles both as basic substrates and as regulators in many metabolic pathways. Interestingly, marked change in the plasma amino acid pattern was detected by GC/TOF-MS analysis in each PCOS phenotype comparing to the control, suggesting the abnormality of amino acids catabolism and biosynthesis (Table 3). We observed that the concentrations of valine and tryotophan were generally elevated in PCOS groups. By contrast, the levels of glycine and proline in four phenotypes of PCOS were decreased significantly compared with the corresponding control group levels. Additionally, higher levels of alanine, serine, threonine, phenylalanine, ornithine and tyrosine were observed in anovulatory PCOS patients. Surprisingly, plasma serine and threonine levels were inhibited in women with PCOS with normal ovulation as compared with controls. Moreover, the concentrations of total endogenous amino acids and gluconeogenic amino acids were markedly increased in the classic and nonhyperandronetic PCOS phenotypes (A and B groups) compared to the control samples, indicating the induced proteolysis and inhibited gluconeogenesis in these women with PCOS. In particular, although both BACC and AAA concentrations were augmented in PCOS samples, the ratio of BCAA to AAA was dramatically reduced in anovulatory PCOS phenotypes as compared with the control group, respectively.

### Effects of obesity and insulin resistance on the metabolic changes in PCOS

Approximately 40% patients with PCOS in our study were obese (BMI ≥25) and 30% were suffered from insulin resistance (Homeostasis Model Assessment of Insulin resistance (HOMA-IR) ≥2.69) [18]. To evaluate the potential interferences of obesity and insulin resistance with the metabolic abnormalities in PCOS, we performed the correlation analysis to test the associations between specific metabolites and PCOS, BMI and IR. Taken together, we observed the significantly positive association of linoleic acid, stearic acid, alanine, serine and tryptophan concentrations with the occurrence of PCOS disease, and the obviously negative relations of glucose, proline and isoleucine levels with PCOS controlling for age, BMI and IR (Additional file 1). However, plasma concentrations of valine, glycine, serine and threonine were closely correlated with IR and obesity (Additional file 2). Additionally, significant positive association of lactate and leucine concentrations with IR was observed independently of obesity. Extraordinarily, significant decreases of serine and threonine levels were observed in both obese women with PCOS and patients with IR comparing to the nonobese PCOS subjects and patients with normal insulin sensitivity, respectively. In contrast, serine and threonine concentrations in PCOS samples were notably enhanced as compared with the normal controls independently of obesity or IR, indicating opposite impacts of PCOS and its two common features on the metabolism of serine and threonine (Additional files 3 and 4).

### Metabolic pathways associated with PCOS

**Figure 3:**
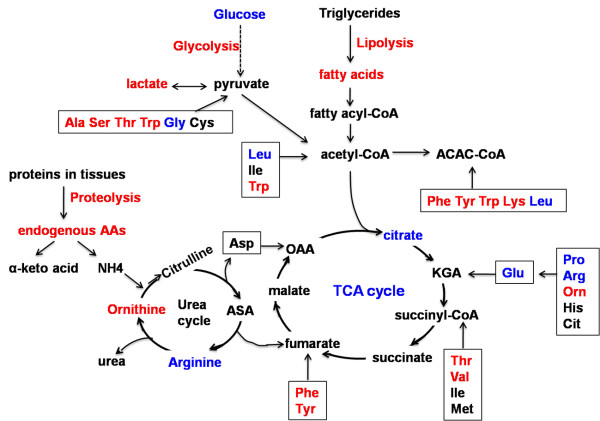
**Metabolic pathways associated with polycystic ovary syndrome (PCOS) development**. The schematic map was modified from [17], with permission from the American Chemical Society (copyright (2007)). Amino acid metabolic pathways are grouped according to their points of entry into the tricarboxylic acid (TCA) cycle, glycolysis, ketogenesis, lipolysis, proteolysis and urea cycles. Glucogenic amino acids can be broken down into one of the following metabolites: pyruvate (Ala, Ser, Thr, Try, Gly, Cys), α-ketoglutarate (KGA) (Pro, Arg, His, Glu, Orn, Cit), succinyl CoA (Val, Thr, Ile, Met), fumarate (Phe, Tyr) or oxaloacetate (OAA) (Asp); while ketogenic amino acids can be broken down into acetoacetyl-CoA (Phe, Tyr, Trp, Lys, Leu) or acetyl-CoA (Ile, Leu, Try). The increase (red) and decrease (blue) of metabolites concentrations in the PCOS plasma were on the basis of both our data and previous report (reduction of arginine and citrate in PCOS patients [19]). The glycolysis, lipolysis and proteolysis pathways were induced in women with PCOS, whereas the TCA cycle and ketogenesis were inhibited in PCOS. Fatty acids included linoleic acid, palmic acid and stearic acid. Amino acids are abbreviated using the standard three-letter convention. AA = amino acids; ACAC-CoA = acetoacetyl-CoA; ASA = argininosuccinate.

With the aid of the Kyoto Encyclopedia of Genes and Genomes (KEGG) database, metabolic pathways associated with PCOS were summarized in Figure 3 based on the changes of intermediates concentrations detected in this study. The affected metabolic pathways in PCOS patients included the tricarboxylic acid (TCA), glycolysis, ketogenesis, lipolysis, proteolysis and urea cycles. Elevated rates of peripheral glucose uptake and diminished hepatic lactate conversion to glucose suggested the enhanced glycolysis in muscle and inhibited gluconeogenesis in liver during occurrence of PCOS. Additionally, the PCOS patients were accompanied by marked impairment of TCA cycle. This was reflected in the reduced citrate level and elevated levels of plasma threonine, valine, phenylalanine and tyrosine, inducing a drop in succinyl-CoA and fumarate. In addition, the women with PCOS advanced quickly to a state of lipolysis and protein catabolism, evidenced by increased levels of plasma fatty acids and amino acids. An elevated level of ornithine and decreased level of arginine implied the imbalance of urea cycle (a reduced level of citrate and arginine has been reported [19]).

## Discussion

The etiology of PCOS is complex, and the present results demonstrated the changes of metabolite profiles in the different PCOS phenotypes, which reflected the metabolic heterogeneity of the PCOS population and offered potential to study the underlying causes. Our findings clearly show that PCOS is associated with aberrations in carbohydrate metabolism. The significant elevation of lactate and glucogenic amino acids and the reduction of glucose in PCOS plasma implied elevated glycolysis in muscle and decreased gluconeogenesis in liver during PCOS pathogenesis. The strongly positive correlation of lactate level to insulin resistance further suggested insulin stimulated glucose uptake and consumption in the muscle of these PCOS patients (Additional files 1 and 4).

In terms of lipid metabolism, subjects with PCOS had higher triglycerides, LDL and VLDL levels and a lower HDL level (Tables 1 and 2), which is consistent with previous reports [20,21] and manifested lipid disorders and dyslipidemia development. Moreover, plasma metabolic profiles in our results indicated the dramatically increased levels of three long-chain fatty acids (palmic acid, stearic acid, linoleic acid) in PCOS samples compared with the controls, irrespective of obesity or insulin resistance (Table 3 and Additional file 1). Previous reports have suggested the levels of linoleic acid in the follicular fluid significantly decreased during follicle size increase in cattle, and linoleic acid supplementation could inhibit bovine cumulus expansion, leading to reduce oocyte maturation and developmental potential [22]. The increase of linoleic acid levels in PCOS plasma may be accompanied by a similar change in the follicular fluid, thus resulting in blocked oocyte maturation and ovulation in PCOS. Additionally, linoleic acid displayed potent proinflammatory activities [23], so the higher level of linoleic acid might be not only linked with increased lipolysis and ovarian dysfunction, but also to the chronic low-grade inflammation in PCOS patients.

Additionally, our study showed different amino acid profiles in PCOS phenotypes for the first time, and the distinct patterns of free amino acids in PCOS and control subjects in the current study provided us important biochemical information and metabolic signatures that enabled the diagnosis of PCOS. More recently, some prospective studies have reported potential amino acid biomarkers for IR and diabetes. Newgard *et al. *[24] reported that circulating concentrations of branched-chain amino acids (Val, Leu, Ile) contributed to development of obesity-associated insulin resistance. Wang *et al. *[25] identified 5 branched-chain and aromatic amino acids (Val, Leu, Ile, Phe, Tyr) from 61 metabolites profiled as the markers of insulin resistance and predictors of the future development of DM2. Further, Wurtz *et al. *[26] reported the alterations in branched-chain and aromatic amino acid metabolism precede hyperglycemia in the general population. In our study, glycine was a novel amino acid we found which was closely related to IR except for valine and leucine, (Additional file 2). Elevated level of valine and reduced level of glycine were also observed in the women with PCOS without insulin resistance as compared with controls, and these changes were further aggravated when the patients had impaired insulin sensitivity (Additional file 4), which underlined valine and glycine were associated with other metabolic disturbances except for IR in the development of PCOS. Additionally, glycine has been shown to improve the proinflammatory profile and upregulate adiponectin gene expression *in vitro *[27]. Adiponectin levels seem to be lower in women with PCOS compared with non-PCOS controls after controlling for BMI-related effects [28]. Consequently, the reduced level of glycine might downregulate the expression of adiponectin and lead to the inflammation in women with PCOS independently of obesity. Thus, glycine could also be useful as a modulator of the inflammatory state observed in PCOS.

Leucine has been shown to rescue insulin signaling via activation of the mTOR pathway, and increasing dietary leucine intake can improve insulin sensitivity and restore many metabolic abnormalities [29,30]. Leucine uptake gradually increases during follicle development, whereas this increasing rate decreases in preovulatory follicles [31]. Considering there was no significant difference of leucine level between women with PCOS with normal insulin sensitivity and control subjects in our results, we speculated that alterations of leucine plasma level in PCOS patients with IR might be entirely due to the impairment of insulin signaling. In terms of aromatic amino acids, the obvious changes of AAA levels and BCAA/AAA ratio were independent of insulin resistance and obesity, which were inconsistent with other findings in DM2 [25,26]. Although IR is a common manifestation of PCOS and women with PCOS have an increased risk of developing DM2, the pathogenesis of PCOS and DM2 were entirely different, which was indicated by these distinct amino acid profiles in women with the two diseases.

Some previous reports have shown the clinical and endocrine disorders in different PCOS phenotypes [32,33]. In this study, the classic phenotype of PCOS was associated with more adverse biochemical and metabolic changes than other phenotypes when compared with controls. Alterations of LH level, LH/FSH ratio, AAA levels and BCAA/AAA ratio were much more severe in classic PCOS than in other PCOS phenotypes (Tables 1 and 3).

Moreover, ovulatory PCOS phenotype had different changes of metabolic profile than the anovulatory PCOS phenotypes. The total concentration of endogenous amino acids was suppressed in ovulatory women with PCOS compared with the control group (Table 3), which demonstrated increased protein synthesis in those patients as Carmina *et al*. had reported that lean muscle mass was increased in women with PCOS [34]. However, the total level of endogenous amino acids was elevated in PCOS patients accompanied with the clinical feature of polycystic ovary and anovulation, indicating elevated protein degradation during ovarian dysfunction. We further noted ovulatory dysfunction of PCOS patients with raised production of the following amino acids: serine, threonine, phenylalanine, tyrosine and ornithine were significantly elevated only in the anovulatory PCOS subgroups, which implied that enhancements of these five amino acids might be directly related to ovulatory dysfunction by their increased ovarian uptake in PCOS patients. The main pathway to *de novo *biosynthesis of serine starts with the glycolytic intermediate 3-phosphoglycerate, so the increase of serine in anovulatory PCOS patients probably arises from increased glycolysis. Specially, the levels of serine and threonine were obviously reduced in ovulatory PCOS subtype, which might be due to the significantly negative correlations of serine and threonine to obesity and insulin resistance (Additional files 3 and 4). The concentrations of these two amino acids were indeed inhibited in PCOS patients with obesity or insulin resistance as compared with PCOS controls, respectively (Additional files 1 and 2). All of these findings together confirmed the metabolic heterogeneity of PCOS due to various clinical features. In addition, the elevated aromatic amino acids levels and decreased BCAA/AAA ratio in anovulatory patients were strongly related to the severity of the PCOS phenotypes (Table 3). In relation to this point, the significantly decreased BCAA/AAA ratio may be considered as a crucial marker of the development of PCOS.

Furthermore, androgen excess was the most common phenotype of PCOS and was somehow associated with insulin resistance. However, we observed different roles of these two phenotypes on metabolic components of PCOS. Androgen excess was closely related to the lipid metabolic disorder, the levels of three long-chain fatty acids (for example, palmic acid, stearic acid, linoleic acid) were all significantly reduced in PCOS patients with hyperandrogenism compared with women with PCOS without clinical or biochemical hyperandrogenism (Additional file 5), whereas no significant difference of these fatty acids levels were observed between PCOS cases with IR and without IR. Additionally, lactate, leucine and glycine were closely related to insulin resistance of PCOS, but the levels of these metabolic components were not influenced by androgen excess of PCOS (Additional files 4 and 5).

Otherwise, our results of metabolic signature in PCOS are partially inconsistent with previous reports. Escobar-Morreale *et al*. found PCOS was associated with decreased alanine concentrations [35], but GC/TOF-MS analysis in our data revealed obviously elevated levels of alanine in PCOS plasma. Alanine is transferred to the circulation mainly by skeletal muscle. There are two main pathways of alanine production: directly from protein degradation, and via the transamination of pyruvate by alanine aminotransferase (ALT). Women with PCOS have been implicated to have higher levels of ALT in the serum [36], which could accelerate the transamination of pyruvate to alanine. Additionally, increased expression of pyruvate dehydrogenase kinase 4 (PDK4) mRNA in PCOS patients [37] can enhance the peripheral concentration of this enzyme and subsequently promote the conversion of pyruvate to lactate, supporting the higher lactate concentration and glycolytic rate in our results. In addition, all control subjects have normal weight and insulin sensitivity, and we need samples from control women with obesity or insulin resistance for comparison to further analyze the effect of obesity and insulin resistance on the metabolic changes in PCOS. Another limitation is that we did not perform the subsequent replication using more samples. We are now enlarging our sample size to confirm these findings and trying to make a possible diagnostic model using the combined values of differentiate metabolites.

## Conclusions

PCOS is a persisting challenge for clinical and basic research to elucidate its origins and distinguish primary pathological changes from secondary environmental disruptions. This study has important significance not only in identifying novel metabolic biomarkers that predict PCOS and its long-term complications, but also in better understanding of the metabolic pathways directly affected during PCOS occurrence, which may provide a rational basis for the development of novel therapeutic targets. Further correlation-based network analysis may help us to generate a diagnostic index of PCOS based on the plasma concentrations of amino acids and other metabolites detected in this study.

## Competing interests

The authors declare that they have no competing interests.

## Authors' contributors

JQ, LF and RL participated in the study concept and design, analysis and interpretation of data, and critical revision of the manuscript. YZ did the statistical analyses and wrote the manuscript. LF, L-NW, YY, YW and PL helped to collect the samples. N-NL and C-MZ completed the clinical information. B-BT and XZ participated in sample preparation. All authors are guarantors of the work. All authors participated in the revision and final approval of the manuscript, and had full access to the data of the study.

## Pre-publication history

The pre-publication history for this paper can be accessed here:

http://www.biomedcentral.com/1741-7015/10/153/prepub

## Supplementary Material

Additional file 1 **Table S1**. Relation of specific metabolites concentrations to polycystic ovary syndrome (PCOS) controlling for age, body mass index (BMI) and insulin resistance (IR). Click here for file

Additional file 2 **Table S2**. Correlation of insulin resistance and obesity to the levels of differential metabolites detected in PCOS plasma. Click here for file

Additional file 3 **Table S3**. Description: Interferences of obesity on the metabolic abnormalities in polycystic ovary syndrome (PCOS). Click here for file

Additional file 4 **Table S4**. Interferences of insulin resistance on the metabolic abnormalities in polycystic ovary syndrome (PCOS). Click here for file

Additional file 5 **Table S5**. Interferences of androgen excess on the metabolic abnormalities in polycystic ovary syndrome (PCOS). Click here for file
