# Supplementary material for: Metabolic profiles characterizing different phenotypes of polycystic ovary syndrome: plasma metabolomics analysis
Source: BMC Med. 2012 Nov 30;10:153. doi: 10.1186/1741-7015-10-153 (PMC3599233; doi:10.1186/1741-7015-10-153)
Supplement: Additional file 1 — Table S1. Relation of specific metabolites concentrations to polycystic ovary syndrome (PCOS) controlling for age, body mass index (BMI) and insulin resistance (IR). [file 1741-7015-10-153-S1.DOC]

**Supplementary Table 1:** Relation of specific metabolites concentrations to PCOS controlling for age, BMI and IR.

**
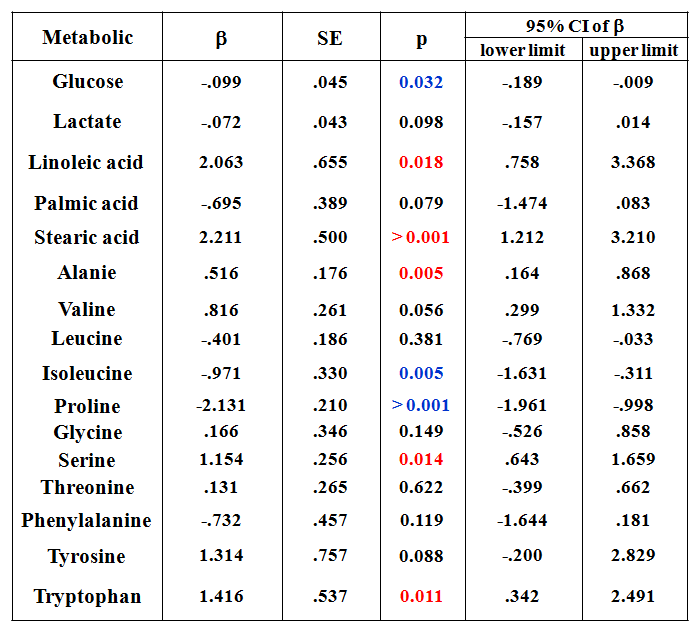
**

**Note:** Regression coefficient (β) shown, from linear regression analysis in the samples controlling for age, BMI and IR. The Red and Blue indicated the significantly positive or negative correlation respectively (P<0.05).
