# Supplementary material for: Metabolic profiles characterizing different phenotypes of polycystic ovary syndrome: plasma metabolomics analysis
Source: BMC Med. 2012 Nov 30;10:153. doi: 10.1186/1741-7015-10-153 (PMC3599233; doi:10.1186/1741-7015-10-153)
Supplement: Additional file 2 — Table S2. Correlation of insulin resistance and obesity to the levels of differential metabolites detected in PCOS plasma. [file 1741-7015-10-153-S2.DOC]

**Supplementary Table 2:** Correlation of insulin resistance and obesity to the levels of differential metabolites detected in PCOS plasma.


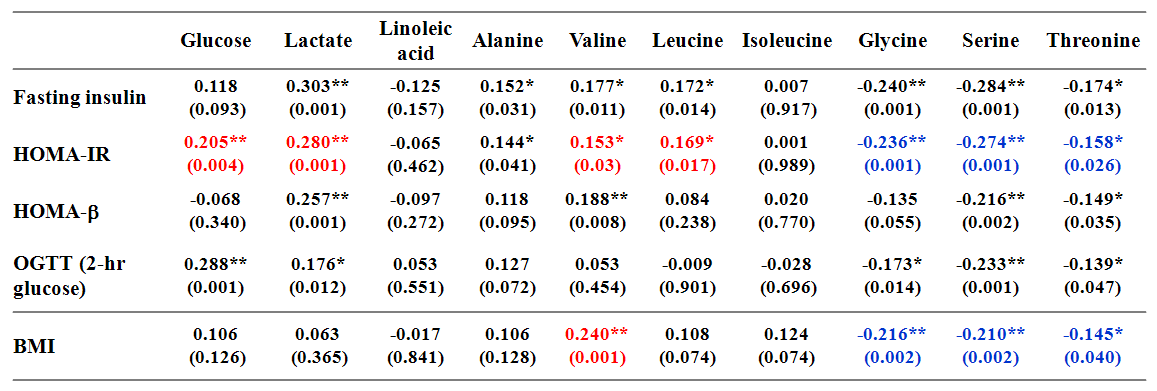


Note: Pearson correlation coefficients (r) shown, from bivariate correlations in the PCOS samples (n=203). Values in parentheses are p-values, the Red and Blue indicated the significantly positive or negative correlation respectively (* P<0.05 , **P<0.01). HOMA-IR: homeostasis model assessment of insulin resistance. HOMA-β: homeostasis model assessment of beta cell function. OGTT: oral glucose tolerance test. WTH: waist to hip ratio.
