# Supplementary material for: Metabolic profiles characterizing different phenotypes of polycystic ovary syndrome: plasma metabolomics analysis
Source: BMC Med. 2012 Nov 30;10:153. doi: 10.1186/1741-7015-10-153 (PMC3599233; doi:10.1186/1741-7015-10-153)
Supplement: Additional file 4 — Table S4. Interferences of insulin resistance on the metabolic abnormalities in polycystic ovary syndrome (PCOS). [file 1741-7015-10-153-S4.DOC]

**Supplementary Table 4:** Interferences of insulin resistance on the metabolic abnormalities in PCOS.


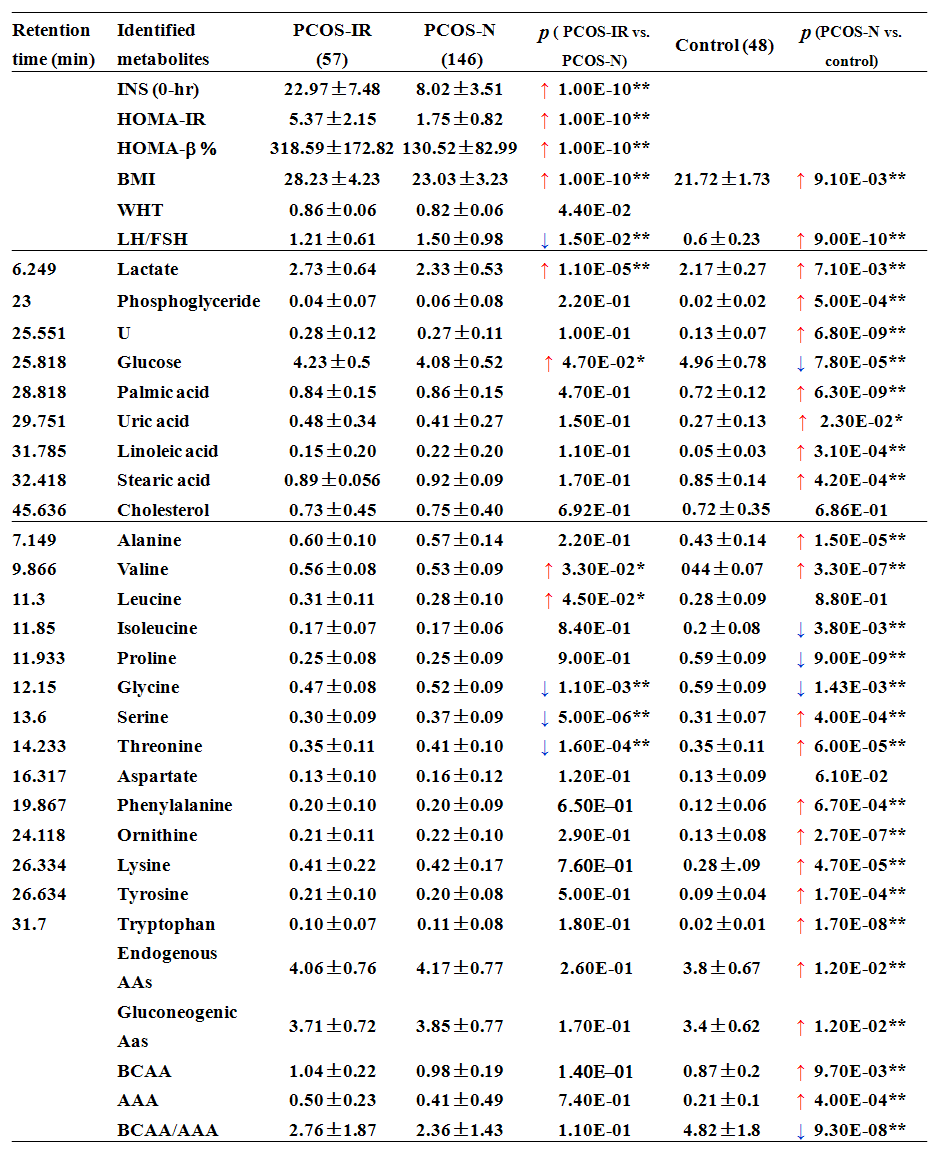


Note: Data are presented as means±SD, and the arrows ↑and ↓ indicated the significant elevated or reduced concentration of metabolites (*P<0.05; **P<0.01) respectively. Endogeneous amino acids include all amino acids except for ornithine; Gluconeogenic amino acids include alanine, valine, isoleucine, proline, glycine, serine, threonine, aspartate, phenylalanine,lysine and tyrosine; BCAA include leucine, valine and isoleucine; AAA include phenylalanine, tyrosine and tryptophan; U, unidentified sugar.
